# Pretreatment of vinasse from the sugar refinery industry under non-sterile conditions by *Trametes versicolor* in a fluidized bed bioreactor and its effect when coupled to an UASB reactor

**DOI:** 10.1186/s13036-016-0042-3

**Published:** 2017-01-23

**Authors:** Elda España-Gamboa, Teresa Vicent, Xavier Font, Jorge Dominguez-Maldonado, Blondy Canto-Canché, Liliana Alzate-Gaviria

**Affiliations:** 1 Yucatan center for scientific research (CICY), Renewable Energy Unit and Biotechnology Unit. Street 43 N.130 Col. Chuburná de Hidalgo 97200, Merida, Yucatan Mexico; 2 Departament d’ Enginyeria Química, Escola Técnica Superior d’Enginyeria, Universitat Autònoma de Barcelona, Bellaterra, 08193 Barcelona, Spain

**Keywords:** Hydrous ethanol vinasse, *Trametes versicolor*, Non-sterile conditions, Fluidized bed bioreactor, UASB, Recalcitrant compounds

## Abstract

**Background:**

During hydrous ethanol production from the sugar refinery industry in Mexico, vinasse is generated. Phenolic compounds and melanoidins contribute to its color and make degradation of the vinasse a difficult task. Although anaerobic digestion (AD) is feasible for vinasse treatment, the presence of recalcitrant compounds can be toxic or inhibitory for anaerobic microorganism. Therefore, this study presents new data on the coupled of the FBR (Fluidized Bed Bioreactor) to the UASB (Upflow Anaerobic Sludge Blanket) reactor under non-sterile conditions by *T. versicolor*. Nevertheless, for an industrial application, it is necessary to evaluate the performance in this kind of proposal system.

**Results:**

Therefore, this study used a FBR for the removal of phenolic compounds (67%) and COD (38%) at non-sterile conditions. Continuous operation of the FBR was successfully for 26 days according to the literature. When the FBR was coupled to the UASB reactor, we obtained a better quality of effluent, furthermore methane content and yield were 74% and 0.18 m^3^ CH_4_/ kg COD_removal_ respectively.

**Conclusions:**

This study demonstrated the possibility of using for an industrial application the coupled of the FBR to the UASB reactor under non-sterile conditions. Continuous operation of the FBR was carried out successfully for 26 days, which is the highest value found in the literature.

## Background

The production of alcohol from sugarcane and its derivatives is a very important industry in Mexico. In 2013, 17 million liters of hydrous ethanol were produced and it will be increased in the next years. A traditional ethanol plant produces between 9 and 14 l of wastewater known as vinasse by liter of ethanol [1]. In general, vinasse is a low pH brown-colored residue that exhibits high concentrations of organic and inorganic compounds. The presence of phenolic compounds (8,000 – 10,000 mg/L), melanoidins (result from the reaction of sugars and proteins by the Maillard reaction), caramel and the furfural components contribute to its color and make vinasse a complex and difficult wastewater for degradation [1, 2].

Vinasse also takes a high value as fertilizer, due to its high organic matter and micronutrients content is often used in crops fertigation. However, when used in large quantities, vinasse can saturate the soil and contaminate nearby water bodies. In Mexico, the application of crude vinasse to sugarcane crops has been observed to affect crop growth without reducing the amount of fertilizer. Thus, it is necessary to dilute vinasse to 10% v/v with water in order to use it in fertigation. Recently, González and Mejía [3] determined the effect of fertigation with vinasse over 50 years on the groundwater quality of an aquifer in the east central region of Mexico. They found that the aquifer has the potential to be contaminated by deep percolation of irrigation water. Therefore, a conditioning treatment must be applied to this wastewater before it is disposed of in the environment.

An anaerobic treatment of vinasse has often been cited as an effective and economical treatment option because it eliminates the chemical oxygen demand (COD) and converts it to biogas, which is a readily usable fuel for the ethanol facility [4, 5]. However, although anaerobic digestion of most types of vinasses is feasible and quite appealing from an energy point of view, the presence of recalcitrant compounds can be toxic or inhibitory for anaerobic microorganism, commonly phenols, melanoidins, and a variety of sugar decomposition products. This slows down the kinetics and reduces mean rates of methane production and yield coefficients [4, 6, 7]. Moreover, the presence of phenolic compounds in drinking and irrigation water represents a health and environmental hazard.

An option for the elimination of phenolic compounds and melanoidins is the use of white rot fungi, which, when cultured under appropriate conditions, produce extracellular enzymes include manganese peroxidase, lignin peroxidases, and laccases that are capable of breaking a lot of different chemical bonds [8]. Laccases catalyze the oxidation of various aromatic compounds, specifically phenolic compounds (*ortho-* and *para-*diphenols, aminophenols and polyphenols), anilines, polyamines and aryl diamines, as well as some inorganic ions while concomitantly reducing molecular oxygen to water [9]. Likewise, it is found in the literature that the laccases are characterized by the presence of copper centers inside their catalytic core. These enzymes contain at least one type-1 copper (T1), which gives the characteristic blue color, together with at least three additional copper ions: one type-2 (T2) and two type-3 (T3) copper ions, arranged in a trinuclear cluster. The oxidation of the substrate occurs at the T1 copper site, and the extracted electrons are transferred through a His-Cys-His tripeptide sequence to T2/T3 site where the reduction of molecular oxygen in water occurs [10–12]; thus has been reported that when small anions are bound in the T2 and T3 copper atoms the internal electron transfer is disturbed and the laccase is inhibited. Also the laccase inhibition may occur through amino acid residue modification, copper chelation or conformational change of the enzyme. Although some substances may inhibit laccase activity, the addition of certain compounds can enhance its efficiency. For example (NH_4_)_2_SO_4_, K_2_SO_4_ and Na_2_SO_4_ have been shown to improve activities of laccase produced by Sinorhizobium meliloti CE52G [12].

*T. versicolor* is a white rot fungus which is able to degrade and/or mineralize a wide range of pollutants resistant to other microorganisms, such as dyes, polychlorobiphenyls (PCBs), polycyclic aromatic hydrocarbons (PAHs), pesticides, pentachlorophenols and endocrine disruptors [13, 14]. *T. versicolor* is a promising option on treating vinasse; nevertheless, for an industrial application, it is necessary to evaluate the performance of vinasse degradation in a bioreactor. Borras et al. [15] developed a continuous process to degrade Gris Lanaset G (real textile dye) and their research team has experience with degradation of other kinds of pollutants, such as endocrine disruptors, pharmaceuticals, urban wastewater [13] and hospital wastewater [16] using *T. versicolor* in pellet form in a fluidized bed bioreactor.

The aim of this study was to develop an integrated treatment involving the use of a fluidized bed bioreactor inoculated with pellets of *T. versicolor* to degrade recalcitrant compounds contained in hydrous ethanol vinasse and later apply an anaerobic treatment in an UASB for biogas production. The performance of this system was compared with anaerobic digestion of fungally-untreated hydrous ethanol vinasse to assess if a fungal pretreatment step was beneficial.

## Methods

### Fungus

*T. versicolor* (ATCC#42530), provided by the Department of Chemical Engineering (Universidad Autónoma de Barcelona, Barcelona, Spain), was used in all experiments. Pellet production was done as previously described by Font et al. [17]. Pellets obtained by this process were washed with sterile distilled water.

### Hydrous ethanol vinasse

The vinasse was collected from “La Gloria” sugar refinery, located in the municipality of Úrsulo de Galván, Veracruz, Mexico. Some average characteristics of this wastewater are shown in Table 1. All parameters were analyzed according to analytical procedures shown below.

**Table 1:** Hydrous ethanol vinasse characterization

Parameter	Value^a^
pH	4.39 ± 0.006
Phenolic compounds^b^	10,834 ± 1476
COD	110,065 ± 11486
SO_4_^2-^	5,300 ± 1416
TN	1,720 ± 217
N-NH_3_	68 ± 9
PO_4_^3-^	415 ± 67

^a^All values except pH are expressed in mg/L

^b^Expressed in gallic acid

## Results and discussion

### Sterile and non-sterile batch fluidized bed bioreactor treatment

#### Phenolic compounds and COD

**Fig. 1:**
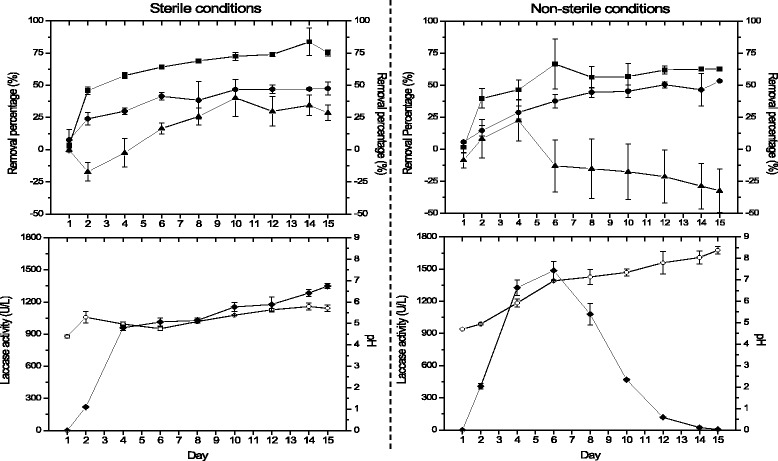
Performance of fluidized bed bioreactor in sterile and non-sterile conditions. Phenolic compound removal (■), COD removal (●), color removal (▲), laccase activity (◆), and pH (○)

The effect of sterile and non-sterile conditions on the performance of bioreactor is shown in Fig. 1. In both conditions it was observed that *T. versicolor* is able to remove phenolic compounds. The initial phenolic concentration on the fluidized bed bioreactor was 1,122 ± 59 mg/L and on day 6 a removal value of 64 ± 0.21% and 67 ± 19% in sterile and non-sterile condition, respectively, was registered. These values remained almost constant until the last day of experimentation (day 15). Similar behavior was registered for COD removal. The initial COD concentration was 12,120 ± 496 mg/L and on day 6 in sterile condition a removal value of 41 ± 3% and in non-sterile condition 38 ± 5% was observed. The removal values, both phenolic compounds and COD, did not show significant difference according to statistical analysis.

The results obtained in both conditions (sterile and non-sterile) are quite interesting since the original phenol content was reduced substantially with a decrease in COD around 40%. A similar result was obtained by García-García et al. [18] during fermentation in batch-wise in a thoroughly mixed reactor using sterilized vinasse using *Aspergillus terreus* and *Geotrichum candidum*. The extent of removal in the fermentation by *A. terreus* of total phenols and o-diphenols was 66 and 94%, respectively; the overall decrease in COD was about 29%. In the case of *G. candidum,* the results were 70 and 91%. The overall decrease in COD, in this case, was about 28%.

#### Laccase activity

Laccase activity presented a different behavior in both conditions: in sterile, laccase registered a value of 960 ± 23 U/L on day 4 and continued to increase slightly until day 15, while in non-sterile condition maximum value was observed on day 6 (1485 ± 86 U/L) after which the activity decreased over time (Fig. 1). An interesting data that can be observed in non-sterile condition is that the phenolic compounds removal remained almost constant although the laccase activity decreased. This suggests the possible existence of other enzymes in *T. versicolor* for phenol degradation. Du et al. [19], support that different oxidative enzymes are involved in the metabolism of *T. versicolor. T. versicolor* F21 shows antagonistic behavior on the cyanobacteria *Microcystis* spp. by degrading the cyanobacteria with extracellular hydrolases and ligninolytic enzymes. They observed that the enzymes cellulose, β-glucosidase, protease and laccase are vital to *Microcystis* spp. degradation in the early stage of incubation (0 h to 24 h) but at 24 h to 60 h appeared manganese peroxidase (MnP). These authors discussed that microcystin can induce oxidative stress in *T. versicolor*, subsequently, the H_2_O_2_-producing enzymes such as glucose oxidase are synthesized in fungal cells to avoid the oxidative damage, and then the degradation reaction of MnP to microcystin is activated by the intracellular H_2_O_2_. This suggests that in non-sterile condition is probable that some compound induced oxidative stress in *T. versicolor* and MnP was produced.

#### Color removal and pH

With respect to color variation and pH, the performance is different in both conditions. In sterile, on day 2 and 4 an increase in color was observed and it was not until day 6 that a removal value of 16 ± 4% was registered, reaching the maximum on day 10 with a removal of 40 ± 14%. On the other hand, in a non-sterile condition, the color variation is large and the average values are in negative values. During sterile condition, the pH was 4.07 on day zero. But, the pH reached 5.6 at day 12 and remained constant until day 15. Likewise, in non-sterile condition the pH was between 4 and 6 during the first 6 days of operation of the reactor and increased to 8 in the following days (Fig. 1).

Table 2 showed several works on vinasse degradation. It was observed that the values found on the literature with respect to color removal are higher than those found in this study due to highest color removal value 40 ± 14% in a sterile condition and color removal in non-sterile condition was not observed. Dahiya et al. [20] said that the better color elimination in sterile condition could have been due to structural changes in compounds when the wastewater was treated at high temperature (121 °C).

**Table 2:** Summary of the vinasse treatment under sterile and non-sterile conditions

Microorganism	Substrate	pH	COD removal (%)	Phenolic removal (%)	Color removal (%)	Reference
*T. versicolor*	Cane molasses vinasse 10% v/v sterile	5.6	41 ± 3	64	16 - 40	This study
*T. versicolor*	Cane molasses vinasse 10% v/v non-sterile	6 - 8	38 ± 5	67	0	This study
*T. versicolor*	Beet molasses vinasse + sucrose and KH_2_PO_4_ sterile conditions not specified	5	77	na	82	Benito et al. [21]
*T. versicolor*	Beet molasses vinasse + sucrose and KH_2_PO_4_ sterile conditions not specified	8	It was not observed	na	10	Benito et al. [21]
*Corioulus hirsutus*	Diluted molasses distillery sterile	4.5	63	na	42	Sun et al. [22]
*Aspergillus-UB2*	Vinasse non-sterile	3	na	na	84	Shayegan et al. [23]
*Trametes pubescens*	Amarulla distillery wastewater sterile	7	73	86	0	Strong [24]
*Corioulus sp.* No. 20	Melanoidins pigments + glucose and sorbose sterile	4.5	na	na	80	Watanabe et al. [27]
*Aspergillus niger*	Beet molasses vinasse y sucrose sterile	na	na	na	45	Miranda et al. [28]

*na* Not available

As can be seen in the Table 2, a lower pH value in the vinasse shows a higher decolorization. Benito et al. [21] evaluated the vinasse treatment with two different pH and reported when the vinasse had a pH value of 8, *T. versicolor* showed almost no growth, COD removal was not produced and only a slight color elimination (10%) was obtained. Figure 1 shows that higher decolorization was in sterile condition. Our bioreactor operated in sterile condition kept a pH value between 4 and 6, unlike non-sterile condition where the pH increased higher to 8. Therefore, it can be suggested that the pH affected decolorization in the fluidized bed bioreactor with non-sterile condition.

Sun et al. [22], reported that increasing the pH in vinasse fungi treatment from 5 to 6 seemed to be unfavorable for laccase activation and is responsible for the decrease in its production. This finding might be attributed to the accumulation of fungal metabolic products in the growing culture which inactivates laccase or inhibits its biosynthesis or the action of proteolytic enzymes. On the other hand, Shayegan et al. [23] mentioned that decolorization can reach a maximum value and then decrease slightly, while the pH increase due to repolymerization of melanoidin. Strong [24] in the treatment of Amarula distillery wastewater (27 g/L of COD and 866 mg/L of phenolic compounds) with *Trametes pubescens* reported that the concentration of phenolic compounds would lead to a great increase in color when the pH increased from 3.8 to neutral. The color change observed may be attributable to the conversion of the phenol to the quinone version in a hydroxyl-rich solution (by abstraction of the hydrogen cation from the OH group of the phenol). Also, it is known that the first step for degradation by fungi is adsorption [24, 25], and pH of the solution affects this step because the fungal cell wall is composed of polysaccharides (i.e., chitin and chitosan), proteins, lipids, and melanin with several functional groups (amino, carboxyl, thiol, and phosphate groups) capable of binding various organic molecules. The ionic forms of these organic compounds in solution and the surface electrical charge of the biomass depend on the pH solution. Therefore, the interaction between a sorbate and sorbent is mainly affected by ionization states of the functional groups on both the molecule and sorbent surface. On the fungi Neurospora intermedia the most suitable pH for sorption is 3, and there was a decrease in the extent of bioadsorption potential with increase in the pH of the solution to values higher than 6 [26].

As can be seen on Table 2, when sugars were added to the vinasse the decolorization was high [21, 27, 28]. Paradoxically, the first studies on the enzymatic system involved in decolorization of vinasse did not focus on ligninolytic enzymes. In fact, intracellular sugar oxidase enzymes were considered as having the most important role in decolorization [29]. The enzyme catalyzing melanoidin decolorization was proved to be L-sorbose oxidase. However, glucose oxidase also decolorized melanoidin pigments. Melanoidin was suggested to be decolorized by the active oxygen (O_2_^-^, H_2_O_2_) produced by the reactions with these oxidases because the reaction with the pure enzyme was accompanied by the oxidation of glucose to gluconic acid [27]. In our study, nutrients like sucrose were not added, the sugar oxidase enzymes were not present during the treatment of vinasse in the fluidized bed bioreactor and that is why the decolorization was lower with respect to the other authors. On the other hand, it was observed induction of laccase activity in the fluidized bed reactor in sterile and non-sterile condition, but no clear correlation with effluent decolorization was observed. This is similar to the report by Yang et al. [30] during dye wastewater treatment, they reached over 80% color removal using white rot fungi but only very low laccase, lignin peroxidase, and manganese dependant peroxidase activities were measured along the whole running process. Thus, no clear correlation about enzymes over decolorization was detected.

#### Final dry mass

**Fig. 2:**
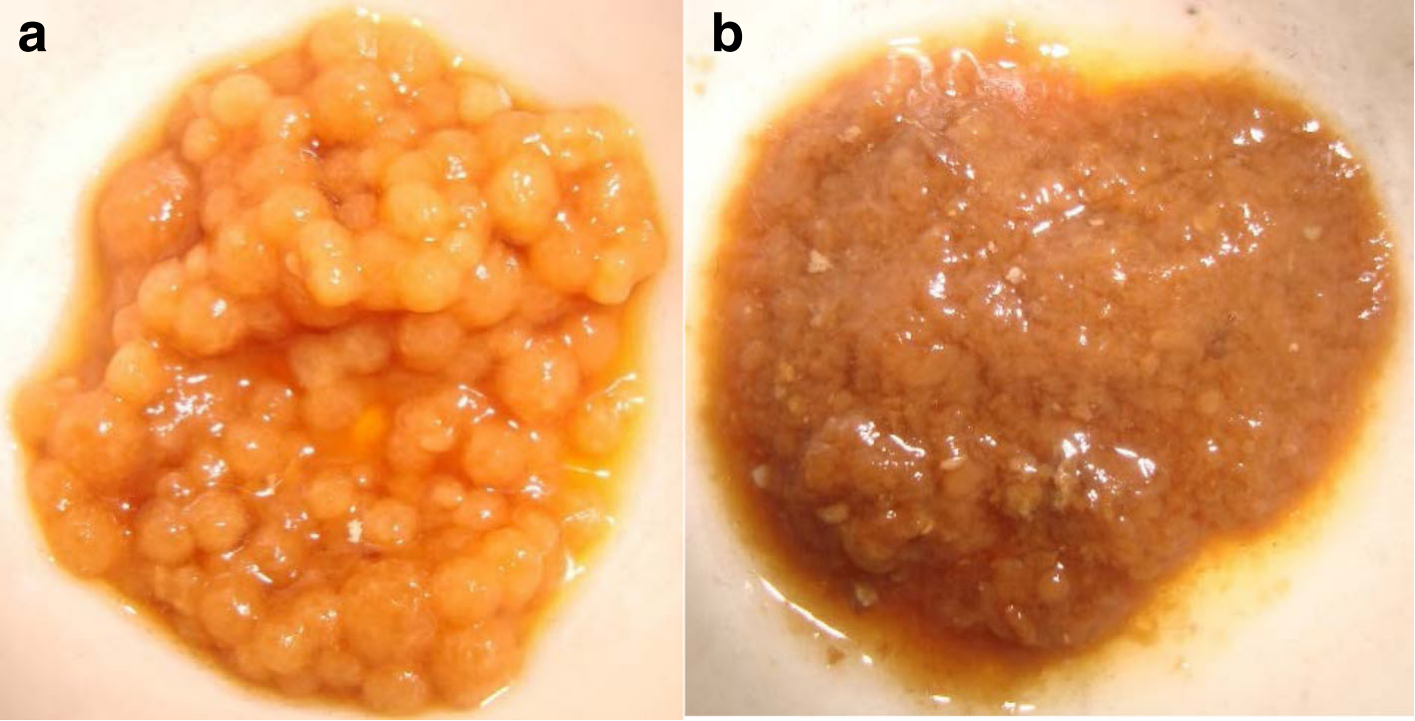
Morphology of pellets of *T. versicolor*: a) Sterile condition b) Non-sterile condition

Final dry mass was measured at the end of both experiments. In sterile condition at day 15 the dry mass was 2.51 ± 0.6 g/L and the morphology of fungal pellets of *T. versicolor* was in the form of compact spherical (Fig. 2a) while at non-sterile condition at day 15 the dry mass was 1.2 ± 0.22 g/L, which was less than initial concentration and the pellets were found disrupted (Fig. 2b). Espinosa-Ortiz et al. [31] mentioned that the robust design of bioreactors to maintain similar performance under sterile and non-sterile conditions is often a challenging task that has not been addressed adequately in the literature, however it was reported that the presence of bacteria on some the fungal system may create competition for the substrate, provoke disruption of the fungal growth, damage the fungal mycelium or reduce the expression of the fungal enzymes, ultimately even leading to the deterioration of the fungal activity.

#### Selection of non-sterile conditions

The phenolic compounds affect severely the anaerobic process and we obtained similar phenolic compound removal in both sterile and non-sterile conditions in FBR; therefore, it was decided to continue the study in non-sterile conditions since this represents an advantage for future industrial application. Especially, significant energy saving may be possible when the production media large volume are directly used without sterilization [32].

### Non-sterile continuous fluidized bed bioreactor treatment

#### Phenolic compounds and COD

**Fig. 3:**
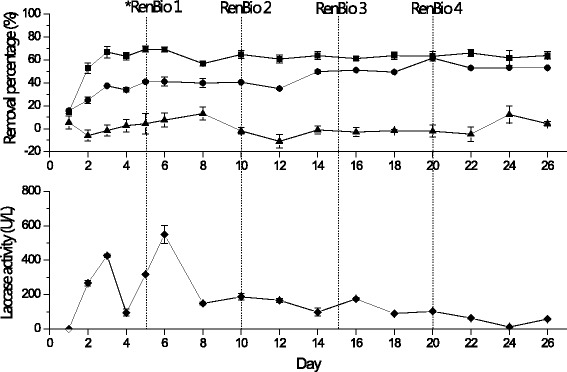
Performance of fluidized bed bioreactor in non-sterile condition operated in continuous. Phenolic compound removal (■), COD removal (●), color removal (▲) and laccase activity (◆).*Ren Bio refers to renovation of T. versicolor biomass inside the bioreactor

In order to obtain feeding for the UASB, the fluidized bed aerobic bioreactor was operated in continuous. Figure 3 shows the performance of the reactor. The initial phenolic concentration in the fluidized bed bioreactor was 1,247 ± 31 mg/L and on day 3 a removal value of 66 ± 5% was registered, and this value remained almost constant until the last day of experimentation (day 26). For COD removal, the initial COD concentration was 10,095 ± 31 mg/L and on day 3 a value of 37% ± 0.8% was observed. This value continued increasing slightly until reaching a value higher than 50%. The COD removal is different as found in batch operation, suggesting that the continuous operation favors the degradation of this parameter.

### Laccase activity

Laccase activity registered a higher value of 550 ± 53 U/L on day 6, which decreased in function of the time. This value did not increase although the fungal biomass was renovated which this is probably due to contamination of the medium with bacteria. The results obtained suggest the possible existence of other enzymes in *T. versicolor* for phenol degradation as it was previously mentioned.

#### Color removal

With respect to color variation, the performance is similar to batch operation due to not observing a high removal value in all the experiment (Fig. 3).

There is little information about vinasse degradation with fungi in reactors operated in continuous mode and most works are focused on the elimination of color and COD. Shayegan et al. [23], during continuous decolorization of anaerobically digested (UASB) distillery wastewater, *Aspergillus-*UB2 was cultivated and transferred to an aeration tank and a maximum level of decolorization of 84% was reached. The efficiency dropped sharply after 96 h. A microscopic examination showed that, due to microbial contamination, the activity of UB2 ceased. It was concluded that it was impossible to work with a reactor for more than a few days and keep the system uncontaminated. In another study, wastewater from a baker’s yeast factory, previously treated by methane fermentation and with activated sludge, was treated in a bubbling column reactor operated in continuous. They reached a decolorization yield of 75%, but maintenance of this condition for a long time could not be stably performed and the decolorization yield decreased gradually after 25 h reaction. Each mycelium becomes small and short because of the bubbling shock and is either washed out from the reactor or undergoes autolysis [33].

Miranda et al. [28] investigated the degradation of vinasse in a jacked tubular reactor. The operation started working as a batch culture and when the color removal was around 40% (3 days), the reactor was continuously fed with wastewater and nutrients determined at a flow rate of 1 L/day (HRT of 5 days). The maximum color removal during the continuous process was 37% and it kept constant for 3-4 days; after this time, the absorbance of the effluent increased, the COD removal was around 69% and the results obtained in the continuous culture indicate a low color removal for a short period of time.

### FBR coupled to the UASB reactor

With the aim of better understanding the discussion of this section, we defined System 1 as the vinasse Untreatment (diluted) affluent to the UASB reactor and System 2 as the fluidized bed bioreactor coupled with UASB reactor. Both systems had a COD of around 7 g/L in affluent to the UASB, because in Mexico, the application of crude vinasse in the crops had been diluted between 7% and 10% v/v with water in order to use it in fertigation. Table 3 shows the characterization of affluent and effluents obtained in systems 1 and 2. According to statistical analysis, the effluent generated in the UASB reactor in each system presented significant differences in all parameters with exception of COD and PO_4_^3-^, suggesting that the UASB effluent of System 2 has better quality for environmental reuse due to lower values of the parameters measured than System 1. Although, there is no significant difference in COD removal of effluent, in Table 3 it can be observed that System 1 had a COD removal of 48 ± 2% while System 2 had a higher total COD removal of 64 ± 3%. This is in accordance to what is reported by Apollo et al. [34] where coupling an ultraviolet photodegradation with an anaerobic digestion process improved the efficiency in COD removal in a real distillery effluent and raw molasses wastewater.

Strong [35], in the treatment of wine distillery wastewater, reported that the fungally-treated samples all had much lower initial color absorbance values and the color increased in a number of the samples after anaerobic treatment of the fungally-pretreated. It is possible that some of the compounds that were depolymerized by the fungal treatment were repolymerized during the anaerobic treatment or possibly due to the increase in pH. In this study, the color in both systems remained constant between affluent and effluent (Table 3).

**Table 3:** Characterization of influent and effluent in systems 1 and 2

	System 1		System 2		
Parameter	Vinasse (diluted 7% v/v)	UASB effluent	Vinasse (diluted 10% v/v)	Fluidized bed bioreactor effluent	UASB effluent
COD	7,704 ± 800	4,000 ± 570^a^	11,000 ± 1,000	7,000 ± 93	3,940 ± 42^a^
Phenolic compounds	758 ± 100	840 ± 42^a^	1,083 ± 150	460 ± 40	519 ± 10^b^
Color*	0.10 ± .001	0.11 ± 0.003^a^	0.13 ± 0.012	0.13 ± 0.003	0.14 ± 0.005^b^
SO_4_^2-^	371 ± 90	200 ± 56^a^	530 ± 141	540 ± 50	70 ± 40^b^
TN	120 ± 15	723 ± 52^a^	172 ± 21	688 ± 79	408 ± 91^b^
N-NH_3_	5 ± 0.6	57 ± 1^a^	7 ± 1	5 ± 0.7	31 ± 4^b^
PO_4_^3-^	29 ± 5	49 ± 6^a^	41 ± 7	22 ± 2	46 ± 7^a^
Biogas production**	-	2,370 ± 149^a^	-	-	1,102 ± 83^b^
Methane content***	-	65 ± 5^a^	-	-	74 ± 6^b^

All values are expressed in mg/L, * Color is the absorbance at 475 nm, ** Biogas production at mL, *** Methane content at percentage. Each value is a mean ± SD. Different case letters indicate significant difference between means of UASB effluent in System 1 and System 2 for each parameter (One-way ANOVA, multiple range test, *P* < 0.05)

**Fig. 4:**
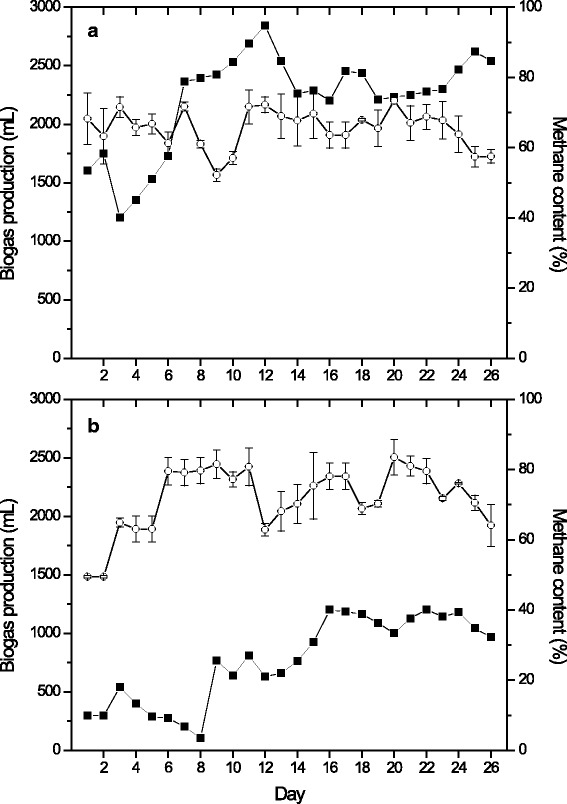
Performance of biogas production and methane content in biogas in: **a**) System 1 and **b**) System 2. Biogas production (■) and methane content (◯)

With respect to phenolic compounds, System 1 did not show a removal value, suggesting that anaerobic digestion in the UASB operated under our conditions is not able to degrade these compounds. Strong [35] detected that the final removal efficiencies of phenol were generally better for anaerobic digestion than for the fungal treatment; however, these removal values were obtained within 2 weeks. In another study, the authors indicated that conversion of phenol and p-cresol is favorable under methanogenic conditions, but a lag phase of 30 days was observed [36]. In our study the UASB was operated with a HRT of 6 days, which is lower than the values reported by other authors for degradation of phenolic compounds. On the other hand, system 2 reached a phenolic compound removal of 52 ± 5%, but it can be detected that the effluent of UASB showed a higher value of phenolic compounds compared with the effluent of fluidized bed bioreactor. This is probably due to cell lysis of pellets of *T. versicolor* that could be mixed in the affluent of the UASB reactor. As it was mentioned previously, the biodegradation for fungi is by adsorption and it is probable that the compounds adsorbed or absorbed by *T. versicolor* were released in the UASB. Figure 4 presents the biogas production and methane content in the biogas for both systems. System 1 registered a higher biogas production (2,370 ± 149 mL) compared with System 2 (1,102 ± 83 mL); however, System 2 reached a higher methane content in biogas (74 ± 6%) than System 1 (65 ± 5%). The statistical analysis showed that these values have significant difference. With these results we found that the methane yield was 0.28 and 0.18 m^3^ CH_4_/ kg COD _removal_ for System 1 and System 2, respectively.

Jiménez et al. [4] reported that the pretreatment of distillery wastewater with *Penicillium decumbens* reduced 67.7% initial concentration of phenolic compounds. Later, they realized anaerobic digestion in a continuous stirred reactor of vinasses with and without treatment with *P. decumbens*. Those authors obtained a methane yield of 0.2 m^3^ CH_4_/kg COD_added_ with a HRT of 53 days and an OLR of 1.5 kg COD/m^3^day with non-treated vinasse; likewise, with the same OLR of treated vinasse, was registered a methane yield of 0.225 m^3^ CH_4_/kg COD_added_ with a HRT of 15 days. Reaching an increase of 12% in the methane yield. In our study, using a ORL similar to Jiménez et al. [4] (1.2 kg COD/m^3^day) were obtained good yields for both systems (0.28 and 0.18 m^3^ CH_4_/kg COD_removed_ for system 1 and 2 respectively) with a HRT of 6 days. However, the methane yield did not increase in system 2 due that a decrease in the biogas production was observed. Because, in system 2 the BOD_5_ in the effluent of FBR inoculated with *T. versicolor* (affluent UASB) was 609 mg/L, suggesting that *T. versicolor* consumed large quantities of the biodegradable organic matter (85% BOD_5_ removal). Similar results were presented by Ferreira et al. [37], who removed 75% of BOD_5_ in sugar cane vinasse when it was treated with *Pleurotus sajor-caju*. Also Kida et al. [38], reported the treatment of Schochu distillery wastewater with *Aspergillus awamori var. kawachi*, they found that the BOD_5_ removal was 56%; therefore, these works showed that a fungus is able to degrade more than 50% of biodegradable organic matter on vinasse.

On the other hand, in the System 1 the BOD_5_ (affluent UASB) was 3,500 mg/L according to a vinasse untreated (approximately 40% of COD) and its value is agree to prior review of the literature [1, 39]. This result suggests that the organic matter available in the UASB reactor was more easily biodegradable and this is reflected in the high biogas flow. However, although the COD was the same in both systems, COD in system 2 could be masked by a greater amount of oxidizable inorganic compounds as nitrites, sulfides, and chlorides found in higher concentration, as shown in Table 3 [40]. Furthermore, the methane content in system 2 was higher, because *Trametes versicolor* was able to remove more than 50% of the phenols present in the vinasse and it has been mentioned previously that these phenolic compounds inhibit to methanogenic microorganisms [4].

Although a lower amount of biogas was obtained in system 2, this presented the best quality to be used in the production of electricity since the common methane content in the crude gas must be at least 65% [41].

## Experimental procedures

### Fluidized bed bioreactor

#### Batch treatment

A glass fluidized bed bioreactor with a useful volume of 2 L, described by Blánquez et al. [42], was used to carry out both sterile and non-sterile vinasse treatment. The vinasse was diluted at 10% v/v with distilled water, given that crops in Mexico are currently irrigated at this concentration. Approximately, 2.4 g dry weight pellets/L were inoculated in sterile and non-sterile treatments. Fungal biomass was maintained with an air flow of 10 L/h generated by an air pump. The pH was not controlled and the temperature was maintained at 25 °C. For sterile conditions the bioreactor and the diluted vinasse were autoclaved at 121 °C for 20 min. Samples of 20 mL were taken daily during the first two days and subsequently each second day during the 15 days of experimentation. The samples were used to measure COD, phenolic compounds, color and laccase activity. The dry weight of mycelium mass was measured (105 °C for 24 h) at the end both experimental conditions.

#### Continuous treatment

The bioreactor was operated in continuous in non-sterile conditions for 26 days. The pH was maintained at 4.5 with a pH controller (ALPHA PH 560, Thermo Scientific). Hydraulic retention time (HRT) was 6 days, which was established based on the result obtained in the batch treatment. The biomass renovation methodology reported by Blánquez et al. [43] was used to extend the operational time. This strategy must allow continuous long-term operation to maintain satisfactory degradation percentages and extracellular enzymatic production. The inoculum, air flow and temperature were the same as in the batch experiment. Samples of 20 mL were taken daily during the first six days and subsequently each second day. The samples were used to measure COD, phenolic compounds, color and laccase activity.

### Upflow anaerobic sludge blanket

#### System 1

A UASB reactor with an operational volume of 4.2 L was operated under mesophilic conditions (30 ± 5 °C) using a water bath WiseCircu®. HRT was 6 days. The reactor was fed daily with 700 mL of ethanol hydrous vinasse diluted at 7% v/v which is equivalent to an organic loading rate (OLR) of 1.2 kg COD/m^3^day. A pH of 7.0 was maintained using sodium bicarbonate (NaHCO_3_) as a buffer. The bioreactor was kept at a minimum operation time of 4 times the HRT (26 days). During the reactor operation, COD, phenolic compounds, color, total nitrogen (TN), ammonia nitrogen (N-NH_3_), phosphate (PO_4_^3-^), and sulfate (SO_4_^2-^) were measured each second day. Gas production and methane content in biogas were measured daily.

#### System 2

The same UASB reactor was operated under identical operational conditions with system 1. The only difference was that it was fed daily with 700 mL vinasse previously treated in a fluidized bed bioreactor with *T. versicolor* equivalent to an OLR of 1.2 kg COD/m^3^-day. This was done in order to find if a fungal pretreatment step was beneficial for the anaerobic digestion. The monitoring of the system was performed identically to system 1.

### Analytical procedures

COD, TN, N-NH_3_, PO_4_^3-^ and SO_4_^2-^ contents were determined via colorimetric methods (Hach Company DR-890), whilst the pH was determined in accordance with American Public Health Association [44].

Phenolic compounds were estimated with Folin–Ciocalteu reagent where 20 μL of sample and 1.58 mL distilled water were placed in an amber-colored flask. 300 μL sodium carbonate solution (20% weight/volume) and 100 μL of, Folin–Ciocalteu reagent were then added in rapid succession, mixed and left to react for 60 min in darkness at room temperature. After the reaction time, absorbance at a wavelength of 765 nm was measured with a spectrophotometer Cole Parmer S2100-UV+ (Cole Parmer, USA). Gallic acid was used as the standard for plotting the calibration curve.

For color determination, the samples were centrifuged at 1,037 *g* for 10 min. They were then diluted to 10% and measured with a spectrophotometer Cole Parmer S2100-UV+ (Cole Parmer, USA) at wavelength of 475 nm [45].

Laccase activity was determined according to Li et al. [46], following the rate of oxidation of 5 mM ABTS (2,2’-azinobis(3-ethylbenzothiazoline-6-sulfonic acid) as a substrate. The measurements were made in 100 mM sodium acetate buffer (pH 5) at 30 °C. Oxidation was measured at 420 nm (ε_420_ = 36,000 M^-1^ cm^-1^). One activity unit (U) was defined as the number of micromoles of ABTS oxidized per minute.

The volume of biogas produced was measured every 24 h with a gasometer (Milligascounter) and biogas methane concentration was determined with a GC Clarus 500-Perkin Elmer equipped with a thermal conductivity detector (TCD) and a Molesieve column, according to España-Gamboa et al. [47].

### Statistical analysis

The optimization experiments were conducted in duplicate. Sampling was done in triplicates and the statistical differences were determined using a one way analysis of variance (ANOVA) with multiple range test using Statgraphics Centurion XVI.

## Conclusions

This study demonstrated the possibility of using a FBR for the elimination of recalcitrant compounds and COD at non-sterile conditions by *T. versicolor*. This shows that significant energy saving may be possible when the production media large volume (industrial application) are directly used without sterilization. Continuous operation of the fluidized bed bioreactor was carried out successfully for 26 days, which is the highest value found in the literature. Two systems were evaluated for the vinasse treatment in which System 2 coupled the FBR to the UASB reactor and registered the best quality of effluent and higher methane content in the biogas.

Conclusively, the coupling of FBR to UASB reactor is a promising environmental technology for the treatment of vinasse; nevertheless, an economic study and feasibility of application of the process to full scale is necessary.

## Abbreviations

ABTS: 2,2’-azino-bis(3-ethylbenzothiazoline-6-sulphonic acid)
ANOVA: Analysis of variance
BOD: Biochemical oxygen demand
COD: Chemical oxygen demand
FBR: Fluidized bed reactor
HRT: Hydraulic retention time
MnP: Manganese peroxidase
N-NH_3_: Ammonia nitrogen
OLR: Organic loading rate
PAHs: Polycyclic aromatic hydrocarbons
PCBs: Polychlorobiphenyls
PO_4_^3-^: Phosphate
SO_4_^2-^: Sulfate
TCD: Thermal conductivity detector
TN: Total nitrogen
UASB: Upflow anaerobic sludge blanket
